# Comparison of sacroiliac CT findings in patients with and without ankylosing spondylitis aged over 50 years

**DOI:** 10.1038/s41598-023-45082-7

**Published:** 2023-10-20

**Authors:** Olivier Fakih, André Ramon, Mickaël Chouk, Clément Prati, Paul Ornetti, Daniel Wendling, Frank Verhoeven

**Affiliations:** 1 Service de rhumatologie, CHU de Besançon, 3 boulevard Fleming, 25030 Besançon Cedex, France; 2 Service de rhumatologie, CHU de Dijon, 14 rue Gaffarel, BP 77908, 21079 Dijon Cedex, France; 3 EA 4267 “PEPITE”, UFR Santé, Franche-Comté University, 19 rue Ambroise Paré, bâtiment S, 25030 Besançon Cedex, France; 4 EA 4266 “EPILAB”, UFR Santé, Franche-Comté University, 19 rue Ambroise Paré, bâtiment S, 25030 Besançon Cedex, France

**Keywords:** Rheumatology, Rheumatic diseases

## Abstract

Diagnosis of axial spondyloarthritis (axSpA) is nowadays commonly made with the help of pelvic radiography or magnetic resonance imaging (MRI). However, there is an important inter-observer variability in radiography, and MRI is subject to possible false positives and is not the best modality for studying structural lesions. Conversely, pelvic computed tomography (CT) has excellent specificity and appears to be more effective than radiography for the diagnosis of ankylosing spondylitis (AS). However, its findings in patients over 50 years of age have not yet been studied. The objectives of this study were to describe the CT characteristics of sacro-iliac joints (SIJ) and the presence of intra-articular gas in patients with AS aged over 50 years and to compare them with controls of the same age and sex. This two-center, cross-sectional, observational study was performed using the medical records of the rheumatology departments of two University Hospitals. We included patients with a clinical diagnosis of axSpA, who had both definite radiographic sacroiliitis according to the modified New York criteria and met the ASAS 2009 criteria for axSpA (that is, AS), and who had undergone any CT scan including the whole SIJ. Each patient was matched for age and sex to a control randomly selected on the Picture Archiving and Communication System (PACS), symptomatic or asymptomatic, and without spondyloarthritis. For each individual, CT scans were interpreted blindly by two independent rheumatologists and scored for joint space narrowing (JSN), erosions, sclerosis, intra-articular gas, and diffuse idiopathic skeletal hyperostosis (DISH). Ninety patients and 90 controls were included in the study. The rates of positive JSN, erosion, and sclerosis scores were higher in the AS group (91% vs. 21%, *p* < 0.0001; 31% vs. 2%, *p* < 0.0001; 27% vs. 13%, *p* = 0.03, respectively), but the rates of intra-articular gas and DISH were higher in the control group (24% vs. 68%, *p* < 0.0001; 7% vs. 33%, *p* < 0.0001, respectively). 58% of patients had complete bilateral ankylosis. A total of 83 (92.2%) patients had a CT scan considered positive for AS, compared with only seven controls (7.8%). Sclerosis and erosions were predominantly on the anterosuperior part and iliac side of the joint in controls and were more diffuse in patients with AS. CT findings in patients with AS over 50 years of age are mostly represented by changes in the joint space; patients with AS have more erosions and sclerosis changes, but less intra-articular gas than controls.

## Introduction

Ankylosing spondylitis (AS) is one of the main chronic inflammatory rheumatic diseases, with a worldwide prevalence of approximately 0.2–0.3%^1^. Diagnosis is based on a combination of clinical, biological, and radiographic findings. The detection of sacroiliitis by conventional radiography or magnetic resonance imaging (MRI) is also part of the classification criteria of the Assessment of SpondyloArthritis International Society (ASAS)^2^.

However, these imaging modalities are limited. For conventional radiography, the main concern is the high inter-observer variability of pelvic radiographs, which does not improve even after training of observers^3^. Several diseases can also mimic sacroiliitis, including diffuse idiopathic skeletal hyperostosis (DISH)^4^.

Magnetic resonance imaging (MRI) has shown interest in detecting inflammatory signs in the sacroiliac joint (SIJ). However, its usefulness in scoring structural lesions, such as erosions, sclerosis, or joint space remains debated^5^. While the EULAR recommendations state that MRI can be used to detect inflammatory and structural lesions^6^, the ASAS definition of a positive sacroiliac MRI focuses on inflammatory signals, without specifically addressing the assessment of structural damage^7^. Moreover, several factors can mimic SIJ inflammatory lesions on MRI, such as early post-partum and exercise^8,9^. Therefore, these lesions must be interpreted with caution because they can lead to a false diagnosis of AS^10,11^.

Conversely, computed tomography (CT) is efficient in evaluating SIJ structural lesions. It enables multi-planar study of all components of the joint and is easily accessible; however, it exposes the patient to ionizing radiation^12^. CT scans have shown superiority over radiographs for the detection of sacroiliitis in patients diagnosed with or consulting for symptoms suggestive of AS^13^. While dedicated SIJ CT is the protocol of choice to evaluate for structural lesions, abdominal CT also has excellent sensitivity and specificity for this purpose^14^. However, these studies did not include control subjects or patients over 50 years of age. To date, CT scans are not routinely used to diagnose AS nor are they included in the ASAS classification criteria, unlike radiography and MRI.

However, in this age group, the main differential diagnosis was SIJ osteoarthritis (OA). Degeneration of the SIJ begins in the third decade of life and becomes more pronounced with age^15^. It is characterized by joint space narrowing (JSN), which is common especially after the age of 50 years, and sclerosis, both of which are also found in AS. Some features seem to be specific to OA, such as the presence of intra-articular gas; however, these changes have not been specifically evaluated in patients with AS. To the best of our knowledge, no study to date has specifically evaluated structural and degenerative lesions of the SIJ on CT scans in patients with AS aged over 50 or compared the prevalence of these lesions in subjects without AS. Therefore, our objective was to describe the prevalence and anatomic distribution of SIJ structural lesions and the presence of intra-articular gas and DISH in AS patients over 50 years of age, and to compare them with age- and sex-matched controls.

## Materials and methods

### Study design and population

All medical records of patients followed in the rheumatology departments of the university hospitals of Besançon and Dijon (France) were screened to identify patients aged at least 50 years, with a clinical diagnosis of axial spondyloarthritis (axSpA), who had both definite radiographic sacroiliitis according to the modified New York criteria^16^ and met the ASAS classification criteria for axSpA^2^ (that is, AS). A search was then carried out in the picture archiving and communication system (PACS) of the two hospitals to identify patients who had undergone a scan to study the sacroiliac joint in its entirety, between the start of archiving (2005) and May 31, 2021. Each patient was then matched with a control of the same sex and age, randomly recruited on PACS, who had also benefited from a scan including SIJs, and who did not meet the criteria for spondyloarthritis (SpA) or have a clinical diagnosis of SpA. Controls also had no history of extra-musculoskeletal manifestations associated with SpA, such as psoriasis, IBD, or uveitis.

Exclusion criteria for both cases and controls were the existence of traumatic or non-traumatic bone lesions and a history of pelvic radiotherapy, which can lead to sclerosis^17^.

For both cases and controls, scans could be of any type (CT of the spine, of the abdomen and pelvis, low-dose whole-body CT, PET-CT, etc.), performed on any machine, and for any reason (low back pain or not), as long as the sacroiliac joints were visible in their entirety and the slice size was less than 3 mm to avoid loss of sensitivity. In the absence of oblique coronal reconstruction on the available images, it was performed by the readers using the viewing software in a standardized manner.

Each CT exam was then scored blindly and independently by two trained and experienced rheumatologists (OF and FV, with 3 and 10 years of experience, respectively). A consensus scoring exercise with a set of 10 randomly selected test cases was used to train readers. These patients were excluded from the analysis.

Patients and controls received information about the use of their medical data and did not object to the study according to the Good Clinical Practice guidelines before the start of the study. This study was performed in compliance with the Declaration of Helsinki and French legislation for the protection of personal data.

### Scoring system and outcome measures

We used a score developed by Poddubnyy et al.^18^ and modified by Diekhoff^19^ to characterize the type and location of structural damage to the SIJ. In this score, each SIJ was divided into 12 regions (four on three reference slices: anterior, middle, and posterior), and for each region, three parameters were numerically scored, namely, JSN, erosions, and sclerosis. For the purposes of this study, we also observed the presence of intra-articular gas and DISH for each region and joint, which are common findings in elderly subjects^20,21^. The presence of intra-articular gas was defined as the presence of a linear gas density in the joint space. DISH was defined as the presence of anterior or posterior bridging osteophytes, with or without associated ankylosis, on both sides of the SIJ. The scoring system used is presented in Table 1.

**Table 1:** Scoring system, modified from Diekhoff et al.^19^.

Joint space		Erosion		Sclerosis		Intra-articular gas		DISH	
0	No joint space change	**0**	No erosions	**0**	No sclerosis	**0**	No intra-articular gas	**0**	No DISH
1	Questionable widening or narrowing	**1**	Small isolated erosions or questionable single erosion	**1**	Questionable or little sclerosis (5–9 mm)	**1**	Intra-articular gas	**1**	DISH lesion
2	Pseudowidening	**2**	Definite erosions or larger single erosion (> 3 mm)	**2**	Evident sclerosis (≥ 10 mm)				
3	Partial ankylosis	**3**	Multiple (> 5) or confluent erosions						
4	Extensive/total ankylosis								

A positive joint space score was defined as a joint space score of 2 or higher, positive erosion score as an erosion score of 2 or higher, and positive sclerosis score as a sclerosis score of 2 or higher in any of the 24 regions studied^19^. A CT scan was defined as positive for AS if there was significant erosion and/or partial or complete ankylosis in the middle or posterior part of the joint^22^. Both readers had to score at least 2 on an item for the lesion to be considered significant in the analyses. Similarly, both readers had to judge whether DISH or gas was present for it to be considered in the analyses. The total score was defined as the sum of the joint space, erosion, sclerosis, intra-articular and DISH scores of the 24 regions studied, thus ranging from 0 to 264.

### Statistical analysis

Quantitative variables are expressed as mean ± standard deviation and were compared using Student's t-test or Mann–Whitney U test, as appropriate. Qualitative variables are expressed as numbers and percentages and were compared using Chi-2 test or Fisher’s exact test, as appropriate. The Kruskal–Wallis test was used to search for a possible imbalance in the anatomical distribution of different lesions. Cohen’s kappa coefficient, simple or weighted if appropriate, was used to assess inter-observer agreement and was interpreted according to Landis and Koch^23^. A value of *p* < 0.05 was considered significant. Analyses were performed using SAS version 9.4 (SAS Institute Inc., Cary, North Carolina, USA).

## Results

After screening the files, 106 scans of the entire sacroiliac joint were found. After excluding patients with traumatic bone lesions (n = 4) and those with a history of pelvic radiotherapy (n = 2), 100 scans were interpreted, including 10 used for the scoring exercise, leaving 90 patients included and analysed in this study, matched to 90 controls. Mean age was 65 ± 10.89 years in the AS group and 65 ± 10.93 years in the control group. There were 62 males (68.89%) in each group. The median time between the scan date of an AS patient and his matched control was 3.0 years (min 0.1–max 14.6 years). In the AS group, 61 patients (85.9%) were HLA-B27 positive. The mean disease duration was 23.0 ± 15.1 years. Twenty-six (41.3%) had a physical profession. Forty patients (44.4%) were currently or previously treated with biologics. Fifteen (17.1%) patients had a history of uveitis, and seven (8.0%) had a history of inflammatory bowel disease. There were 53 former and current smokers (68.0%). The reasons for CT referral in the control group are presented in Table 2. In almost half of the cases, controls underwent CT scans for infection, cancer diagnosis or re-evaluation. Low back pain was the reason motivating the exam in 16 (17.8%) of the controls.

**Table 2:** Referral reasons for CT in the control group.

Referral reason	n (%)
Hip pain	2 (2.2%)
Myeloma screening	5 (5.5%)
Vascular	6 (6.7%)
Abdominal pain	8 (8.9%)
Diffuse pain/polyarthralgia	11 (12.2%)
Low back pain	16 (17.8%)
Cancer diagnosis or monitoring	19 (21.1%)
Infection screening	23 (25.6%)

A comparison of the CT characteristics of the patients with AS and controls is presented in Table 3. Overall, individual lesion positivity was significantly more frequent in the AS group than in the control group. Conversely, intra-articular gas and DISH were found to be significantly more frequent in controls. When comparisons were restricted to the subgroup of patients with AS without total or partial ankylosis (n = 23), there were still differences in the rates of erosion (52.2% in patients vs. 0% in controls, *p* < 0.0001) and sclerosis (30.4% in patients vs. 4.4% in controls, *p* = 0.02), but there was no longer a significant difference in the rate of intra-articular gas (60.9% in patients vs. 73.9% in controls, *p* = 0.35) or in the rate of DISH (17.4% in patients vs. 34.8% in controls, *p* = 0.17).

**Table 3:** Comparison of CT findings in patients in the AS and control groups.

Finding	AS patients	Controls	*p*-value
Mean (SD) total score (range 0–264))	77.74 ± 32.72	15.58 ± 13.67	< 0.0001
Positive CT, n (%)	83 (92.2%)	7 (7.8%)	< 0.0001
Bilateral ankylosis, n (%)	52 (57.8%)	1 (1.1%)	< 0.0001
Positive joint space score, n (%)	82 (91.1%)	19 (21.1%)	< 0.0001
Positive erosion score, n (%)	28 (31.1%)	2 (2.2%)	< 0.0001
Positive sclerosis score, n (%)	24 (26.7%)	12 (13.3%)	0.03
Intra-articular gas, n (%)	22 (24.4%)	61 (67.8%)	< 0.0001
DISH, n (%)	6 (6.7%)	30 (33.3%)	< 0.0001

Positive CT for ankylosing spondylitis is defined by presence of significant erosion and/or partial or complete ankylosis in the middle or posterior part of the sacro-iliac joint.

**Figure 1:**
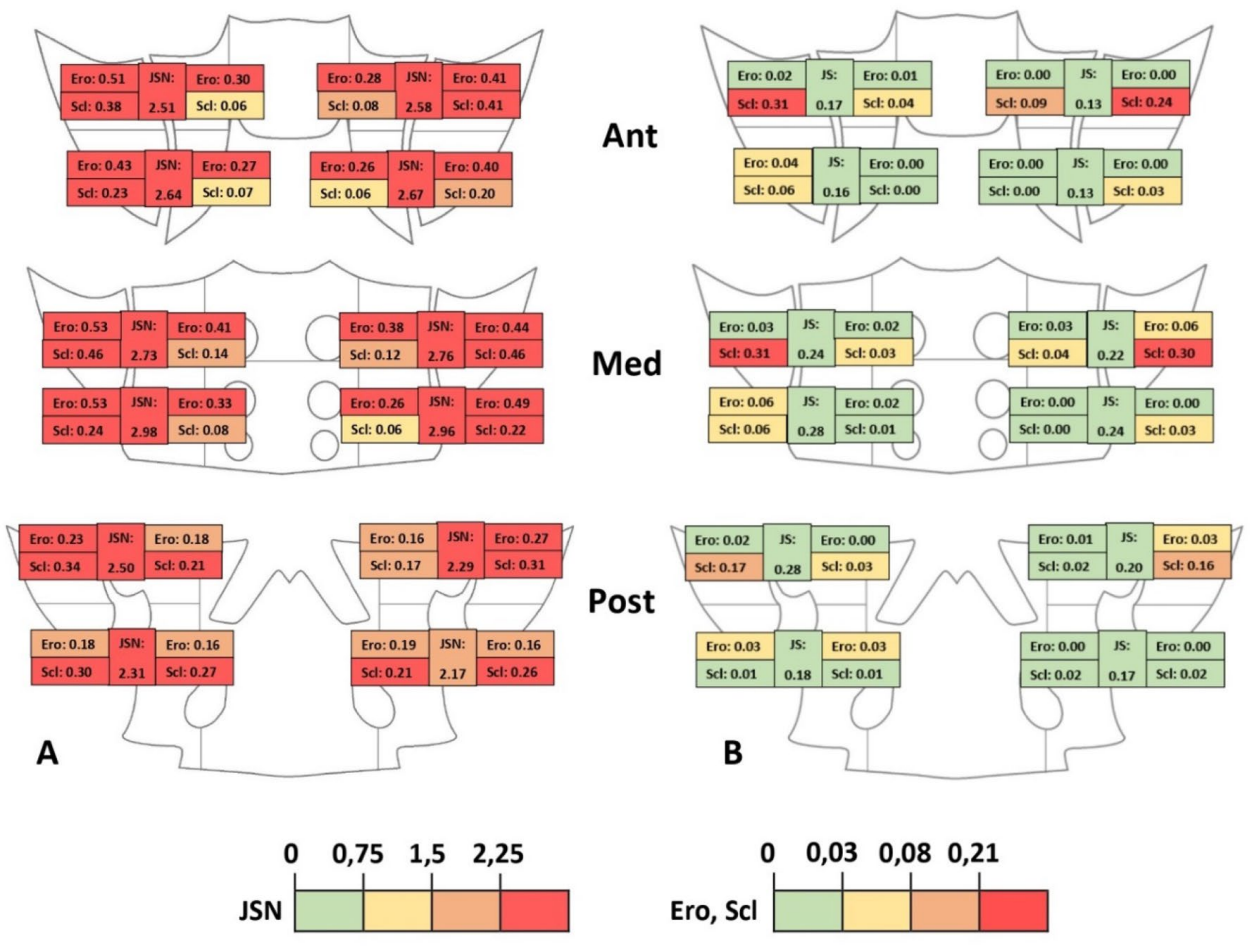
Mean scores by region on anterior, middle, and posterior slices (JSN: joint space narrowing [range 0–4], Ero: erosion [range 0–3], Scl: sclerosis [range 0–3]) in patients with AS (**A**) and controls (**B**).

The means of each score by region for the patients and controls are presented in Fig. 1. In patients with AS, there were significant differences in the anatomical distribution of the JSN (*p* = 0.01), erosions (*p* < 0.0001), and sclerosis (*p* < 0.0001). In the control group, there were significant differences in the distribution of erosion (*p* = 0.04) and sclerosis (*p* < 0.0001) but not in JSN (*p* = 0.80). Comparisons to investigate which factors were associated with structural lesions in the AS group showed that the mean total score seemed higher in patients with a disease duration ≥ 10 years or without intra-articular gas (Table 4). Cohen's kappa coefficient for the analysed patients was 0.88 for joint space assessment, 0.71 for erosions, 0.62 for sclerosis, 0.75 for intra-articular gas, and 1 for DISH.

**Table 4:** Comparison of total scores by different characteristics in patients with ankylosing spondylitis.

Characteristics	n (%)	Mean total score if present	Mean total score if non present
Age ≥ 70 years	29 (32.2%)	55.0	41.0
Disease duration ≥ 10 years	68 (75.6%)	50.1	31.3
Male sex	62 (68.9%)	48.7	38.4
HLA-B27	61 (85.9%)	37.1	29.6
Smoking	53 (68.0%)	42.9	32.2
Physical profession	26 (41.3%)	35.5	29.6
History of inflammatory bowel disease	7 (8.0%)	52.4	43.8
History of uveitis	15 (17.1%)	47.4	43.9
Treatment with NSAID(s)	67 (77.0%)	45.3	39.7
Treatment with biologic(s)	40 (46.0%)	42.6	47.8
Intra-articular gas	22 (24.4%)	21.1	53.4
DISH	6 (6.7%)	24.8	47.0

Total score is defined by the sum of joint space, erosion and sclerosis scores of each region of both sacro-iliac joints for each patient.

## Discussion

This study is the first to describe the CT characteristics of the SIJ in patients with AS aged > 50 years and to compare them with matched controls without AS.

In patients with AS, significant JSN was observed in the vast majority of cases. This finding was consistent with the inclusion criteria of the patients. The erosion rate was lower, but this could be explained by the fact that bilateral ankylosis was found in 58% of cases. Structural lesions were found in a few controls, the most frequent being JSN. Only one case of bilateral ankylosis was found in the context of severe DISH. Only seven of 90 (7.8%) controls met the definition of a positive scan for AS, compared with > 90% of patients. This validates the definition proposed by Hermann et al. for total or partial ankylosis or significant erosion of the middle and posterior parts of the joint^22^.

The intra-articular gas and DISH rates were significantly higher in the control group than in the control group. However, comparisons in the subgroup of patients without total or partial ankylosis were not significant, suggesting that the differences could be explained by the high rate of ankylosis in patients with AS. These results should be interpreted with caution because of the small sample size (n = 23). Overall, erosions remain the most discriminating lesion outside the context of ankylosis. Therefore, CT may be of interest in AS because it is more sensitive than MRI and conventional radiography for the detection of SIJ erosions^13^.The high ankylosis rate may be explained by the high mean disease duration in the AS group.

We also found an uneven anatomic distribution of the different lesions, except for the JSN in the controls. In patients with AS, JSN and erosion were predominant in the anterior and middle parts of the SIJ. It seems that sclerosis predominated in controls on the upper, particularly the anterior and middle parts of the joint, whereas it was diffuse in patients with AS. In addition, it only affected the iliac side of the joint, whereas it also affected the sacral side in patients with AS. Thus, in elderly subjects, sclerosis located in the posterior part or affecting both sides of the SIJ is suggestive of AS. This also allows to not be confused by anterior ankylosis related to DISH.

Our results can be compared to those of a similar study by Hermann et al.^22^. Using the same score to compare CT scans of axial spondyloarthritis patients of any age with those of controls with or without low back pain. The rates of significant erosion and sclerosis in patients with AS were higher than those in the present study. This may be explained by the high proportion of complete bilateral ankylosis linked to older age of the subjects (mean age, 65 vs. 37 years). Similarly, sclerosis and erosions were more frequent on the anterior part of the SIJ, as in our study. This underlines the importance of studying the middle and posterior parts of the joint to differentiate between SA and degenerative changes.

The rates in our control group were similar to those reported in other studies. Eno et al. found a prevalence of degenerative changes > 68% and substantial degenerative changes > 29% in 373 asymptomatic American subjects aged ≥ 50 years, and the proportion increased with age^24^. A study by Bäcklund et al.^25^ based on 246 subjects also showed a high prevalence of degenerative changes, without differences between symptomatic and asymptomatic individuals. Shibata et al.^15^ observed 100% JSN in asymptomatic Japanese subjects over 50 years of age but with a different scoring method.

Regarding DISH, data in the literature concerning its prevalence in the SIJ are sparse. The prevalence of DISH could be as high as 25% for the whole spine^26^, which is still lower than the 30% prevalence observed in our control group. There is no precise definition of DISH in the SIJ, and the distinction between a simple osteophyte related to osteoarthritis sometimes remains subjective.

It should be remembered that there may be a recruitment bias in our control group since all controls underwent a CT scan in a university hospital. Indication bias was limited by the random selection of controls, with no criterion regarding the reason for CT. The prevalence of low back pain is estimated to be between 20 and 75% in individuals aged 60 or more^27^. In our study, 17.8% of CT scans were performed for this reason, but there may be a classification bias, as we did not know whether controls receiving a CT scan for another reason (e.g., to check for infection) had low back pain. To the best of our knowledge, no patients in the control group were known to have crystal arthropathy, which could cause sacroiliitis. Furthermore, exclusion of extramusculoskeletal manifestations limited the risk of including undiagnosed cases of AS in the control group.

Our study has several strengths. It is based on a score widely used in the literature^18,19,22,28^, with good inter-observer agreement for all parameters, even perfect for DISH. Two trained experts read the CT scans. Cohen's kappa coefficient was lower for sclerosis, consistent with what has been found in other studies^19,28^. Thus, this score confirmed its relevance in assessing SIJ structural lesions on CT.

However, our study had several limitations. This was a cross-sectional study using medical records, although few data concerning patient characteristics were missing. Additionally, CT scans could be of any type. Some of these, such as PET-CT or low-dose body-CT, may potentially lack the sensitivity to detect some structural lesions. However, we set a cut-off for a slice thickness of 3 mm to limit errors due to lack of resolution. In addition, we did not consider anatomical variations in SIJs, which can cause degenerative lesions^28,29^, which can vary according to ethnicity^30^, which limits the generalizability of these results.

In conclusion, the CT characteristics of the SIJ in AS patients older than 50 years are mainly represented by bilateral ankylosis and erosions, predominantly on the anterior and middle parts of the SIJ, but with less intra-articular gas and less DISH compared to the control group. These features, especially erosions and intra-articular gas, may help distinguish AS from degenerative SIJ lesions. This study, like others, demonstrates the value of CT scanning for the study of structural lesions in AS, including in an elderly population. Although there is less diagnostic doubt than in a young population, the place of CT scan in the diagnostic approach to AS needs to be studied further, although studies have already been carried out on this subject^31^. In addition, new techniques such as low-dose CT or dual-energy CT could also contribute to the development of the use of this examination in AS^32^.

Moreover, with the increasing availability of targeted therapies slowing the structural progression of the disease, it would be interesting to repeat a similar study in a few years to see whether the rate of ankylosis and erosion in elderly patients is lower than in this study, carried out in subjects who had not been able to benefit the most from these treatments at the time of their diagnosis.

## Data Availability

The datasets used and/or analysed during the current study are available from the corresponding author on reasonable request.
